# A systematic review and meta-analysis of acceptance- and mindfulness-based interventions for DSM-5 anxiety disorders

**DOI:** 10.1038/s41598-021-99882-w

**Published:** 2021-10-14

**Authors:** Heidemarie Haller, Pascal Breilmann, Marleen Schröter, Gustav Dobos, Holger Cramer

**Affiliations:** Department of Internal and Integrative Medicine, Evang. Kliniken Essen-Mitte, Faculty of Medicine, University of Duisburg-Essen, Essen, Germany

**Keywords:** Psychology, Health care, Medical research

## Abstract

This meta-analysis systematically reviewed the evidence on standardized acceptance-/mindfulness-based interventions in DSM-5 anxiety disorders. Randomized controlled trials examining Acceptance and Commitment Therapy (ACT), Mindfulness-Based Cognitive Therapy (MBCT), and Mindfulness-Based Stress Reduction (MBSR) were searched via PubMed, Central, PsycInfo, and Scopus until June 2021. Standardized mean differences (SMD) and 95% confidence intervals (CI) were calculated for primary outcomes (anxiety) and secondary ones (depression and quality of life). Risk of bias was assessed using the Cochrane tool. We found 23 studies, mostly of unclear risk of bias, including 1815 adults with different DSM-5 anxiety disorders. ACT, MBCT and MBSR led to short-term effects on clinician- and patient-rated anxiety in addition to treatment as usual (TAU) versus TAU alone. In comparison to Cognitive Behavioral Therapy (CBT), ACT and MBCT showed comparable effects on both anxiety outcomes, while MBSR showed significantly lower effects. Analyses up to 6 and 12 months did not reveal significant differences compared to TAU or CBT. Effects on depression and quality of life showed similar trends. Statistical heterogeneity was moderate to considerable. Adverse events were reported insufficiently. The evidence suggests short-term anxiolytic effects of acceptance- and mindfulness-based interventions. Specific treatment effects exceeding those of placebo mechanisms remain unclear. Protocol registry: Registered at Prospero on November 3rd, 2017 (CRD42017076810).

## Introduction

Anxiety disorders differ from normative fear or anxiety by featuring exaggerated symptoms lasting persistently over a prolonged period of time that interfere with daily activities. The Diagnostic and Statistical Manual of Mental Disorders, Fifth Edition (DSM-5) classifies anxiety disorders as Separation Anxiety Disorder, Selective Mutism, Social Anxiety Disorder, Panic Disorder, Agoraphobia, Specific Phobias, and Generalized Anxiety Disorder^1^. Globally, the one-year prevalence of anxiety disorders ranges from 2.4% to 29.8% with a point prevalence of 7.3%^2^, while subthreshold anxiety cases are even more common^3^. For most anxiety diagnoses, women are twice often affected than men^4,5^. Patients with anxiety disorders report high rates of co-morbidity^6^ and often suffer from disturbed sleep, headaches, depressed mood, gastrointestinal or cardiovascular problems^7,8^ leading to increasing costs of health care utilization and work loss^9^.

As the first-line treatment of anxiety disorders, clinical practice guidelines recommend psychological therapies, particularly Cognitive Behavioral Therapy (CBT) in preference to or in combination with pharmacotherapy^10–12^. Another treatment option with promising evidence for alleviating anxiety symptoms in non-psychiatric samples^13–17^ are mindfulness-based interventions such as Mindfulness-based Stress Reduction (MBSR), Mindfulness-Based Cognitive Therapy (MBCT), and Acceptance Commitment Therapy (ACT). MBSR is a standardized group program of 8 weekly sessions lasting an average of 2.5 h combined with an additional silent retreat day. Core components of MBSR include sitting and walking meditation, yoga asanas, and mindful relaxation techniques. Daily home practice is demanded to integrate mindfulness into everyday life^18^. MBCT combines mindfulness elements with cognitive-behavioral methods like psychoeducation, cognitive restructuring, and the development of pleasant activities – also within an 8-week group setting including a retreat day and daily home practice^19^. ACT combines acceptance-based and mindfulness strategies with cognitive-behavioral techniques and focuses on accepting experiences while being present, choosing goals according to values, and then taking committed action. ACT is usually an individual-based approach, but also offers group concepts mostly for non-clinical populations^20^. For patients with manifest anxiety disorders, previous systematic reviews of MBSR, MBCT, and ACT with and without meta-analysis suggest significant greater effects in comparison to usual care and comparable effects to CBT but used outdated methodology or focused on single anxiety disorders, mixed anxiety/obsessive–compulsive and depressive syndromes, or elderly patients/children^21–26^. To date, there is no comprehensive meta-analysis that assesses and compares the effectiveness and safety of standardized MBSR, MBCT, and ACT in the management of adult patients with DSM-5 anxiety disorders.

## Methods

The systematic review was conducted in accordance with the Preferred Reporting Items for Systematic Reviews and Meta-Analyses (PRISMA) guidelines^27^ and the Cochrane recommendations^28^. Before starting the review, the protocol was registered at Prospero (CRD42017076810).

### Study selection

Types of studies: Randomized controlled trials (RCTs) or randomized crossover trials were eligible.

Types of patients: Eligible samples included adults diagnosed with an anxiety disorder as defined by DSM-5^1^ including:

- Separation Anxiety Disorder (DSM-5: 309.21/ICD-10: F93.0),
- Selective Mutism (DSM-5: 321.23/ICD-10: F94.0),
- Specific Phobias (DSM-5: 300.29/ICD-10: F40.218, F40.228, F40.23x, F40.248, F40.298),
- Social Anxiety Disorder (DSM-5: 300.23/ICD-10: F40.10),
- Panic Disorder (DSM-5: 300.01/ICD-10: F41.0),
- Agoraphobia (DSM-5: 300.22/ICD-10: F40.00),
- Generalized Anxiety Disorder (DSM-5: 300.02/ICD-10: F41.1),
- Other Specified (DSM-5: 300.09/ICD-10: F41.8), and
- Unspecified Anxiety Disorder (DSM-5: 300.00/ICD-10: F41.9).

Patients who were diagnosed by prior versions of the DSM/ICD were also eligible, if their diagnosis is listed as a DSM-5 anxiety disorder as well. Studies involving patients with anxiety comorbid with other physical/mental disorders were eligible, if the comorbidity was not examined as the primary study outcome. Studies including heterogeneous psychiatric populations such as patients with anxiety disorders as well as those with depression or obsessive–compulsive disorder were excluded, while studies assessing mixed anxiety diagnoses (as defined above) were considered as well.

Types of interventions: Standardized acceptance- or mindfulness-based interventions like MBSR, MBCT, ACT, and variations of these programs (regardless of program length, frequency, or setting) were eligible. Studies allowing individual co-interventions were eligible as long as all participants in all groups received the same co-interventions. Acceptable control interventions were no treatment/wait-list or treatment as usual (TAU) as well as any other active treatments.

Types of outcomes: Improvement in the severity of anxiety symptoms measured by validated clinician- and/or patient-rated scales closest to 2 months after randomization (short-term) were defined as primary outcomes. Secondary outcomes included anxiety symptoms closest to 6 months and 12 months after randomization and safety. The severity of depressive symptoms and health-related quality of life were included as secondary outcomes as well, as anxiety disorders have high rates of comorbidity with depressive symptoms and reduced quality of life. If an outcomes was assessed by more than one instrument, primary endpoints were preferred over secondary ones, disease-specific instruments over generic ones and multi-item over single-item ones. Safety was assessed as the number of adverse events (AE) or study withdrawals due to AEs. AEs were defined as any untoward medical occurrence in a patient, without necessarily having a causal relationship with the study treatment. Cases of any untoward medical occurrence that had resulted in death, was life-threatening, required inpatient hospitalization, or caused persistent or significant disability were rated as serious AEs^29^.

### Data sources

PubMed, Central, PsycInfo, and Scopus were searched from their inception to June 22nd, 2020. An update was executed in PubMed until June 14th, 2021. Table 1 shows the search strategy in PubMed, which was adapted for each database as necessary. No language restrictions were applied. Moreover, we manually searched reference lists of previous reviews. For ongoing and unpublished studies, we searched international trial registries of the WHO and the NIH applying identical search terms. Two reviewers (HH and PB) independently screened titles and abstracts and assessed full-texts for eligibility using EndNote. Any disagreement were rechecked with a third reviewer (HC) until consensus was achieved.

**Table 1:** Search strategy in PubMed.

#1	MBSR[tiab] OR MBCT[tiab] OR mindful*[ tiab] OR Mindfulness[mesh] OR meditation[tiab] OR Meditation[mesh] OR acceptance-based[tiab] OR (acceptance[tiab] AND commitment[tiab] OR Acceptance and Commitment Therapy [mesh])
#2	Anxiety[mesh] OR Anxiety Disorders[mesh] OR anxiety[tiab] OR Phobic Disorders[mesh] OR Phobia, Social[mesh] OR phobia[tiab] OR phobic[tiab] OR Panic Disorder[mesh] OR panic[tiab] OR Agoraphobia[mesh] OR agoraphobia[tiab]
#3	Randomized Controlled Trial[mesh] OR Randomized Controlled Trial[pt] OR Controlled Clinical Trial[pt] OR randomized[tiab] OR randomised[tiab] OR RCT[tiab] OR (random*[tiab] AND allocat*[tiab]) OR (random*[tiab] AND assign*[tiab]) OR placebo*[tiab] OR sham[tiab] OR group[tiab] OR trial[tiab]
#4	#1 AND #2 AND #3

### Data extraction

Two reviewers (PB and MS) independently extracted predefined data on the study setting and characteristics of the included studies. All Discrepancies were rechecked with a third reviewer (HH) until consensus was achieved.

For each study, the risk of selection bias, performance bias, detection bias, attrition bias, reporting bias, and other source of bias were independently assessed by two reviewers (PB and MS) using the Cochrane risk of bias tool^28^. Each domain was assessed as either, ‘low risk of bias’ if all requirements were adequately fulfilled, ‘high risk of bias’ if the requirements were not adequately fulfilled, and as ‘unclear risk of bias’ if insufficient data for a judgment was provided. Divergent assessments were rechecked with a third reviewer (HC) until consensus was achieved.

### Data synthesis

#### Overall analyses

If at least two studies had assessed the same outcome to the same defined time point with the same type of control intervention, pairwise meta-analyses using random-effects models (inverse variance method) with Hedges’ correction for small samples^28^ were conducted using Review Manager Software (RevMan, Version 5.3, The Nordic Cochrane Centre, Copenhagen). For continuous outcomes, standardized mean differences (SMDs) with 95% confidence intervals (CIs) were calculated indicating the difference in means between groups divided by the pooled standard deviation (SD). In cases where no SDs were published, they were calculated from standard errors, CIs or t-values^28^, or were requested from trial authors by email. A negative SMD indicated greater effects of the experimental intervention over the respective control condition, except for quality of life. In accordance with Cohen’s categories, SMDs of 0.2–0.5 were interpreted as small effects, SMDs of 0.5–0.8 as medium effects, and SMDs > 0.8 as large effects^30^. For binary outcomes such as AEs, risk ratio analyses were planned. However, as most studies did not report AEs systematically, AEs were analyzed descriptively.

#### Assessment of statistical heterogeneity

Statistical heterogeneity between the study effects was analyzed by the Chi^2^ statistics with a p-value of ≤ 0.10 indicating significant heterogeneity. The magnitude of heterogeneity was categorized by the I^2^ with I^2^ > 25%, I^2^ > 50%, and I^2^ > 75% representing moderate, substantial, and considerable heterogeneity, respectively^28,31^.

#### Subgroup analyses

Subgroup analyses were planned for patients with different anxiety diagnoses and different mindfulness interventions but could only be realized for the latter, as the number of studies for comparisons was too small.

#### Sensitivity analyses

We conducted sensitivity analyses, where studies with high or unclear risk of bias were compared with those of low risk of bias. If substantial or considerable statistical heterogeneity was present in a meta-analysis, we used sensitivity analyses to explore heterogeneity in effect estimates.

#### Risk of bias across studies

The assessment of publication bias was planned by visual analysis of funnel plots and Egger's test, if more than 10 studies were included in a meta-analysis^32^.

## Results

### Literature search

**Figure 1:**
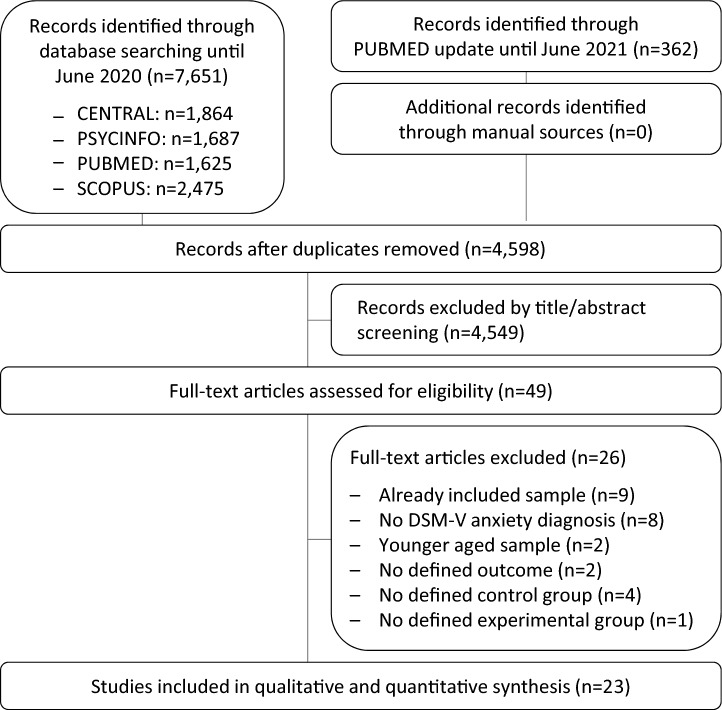
PRISMA flow chart.

The electronic search revealed 7651 articles, a PubMed update additional 362 ones (Fig. 1). After the exclusion of supplicates and non-eligible abstracts, 49 articles were red in full. We excluded further 9 ones as they reported data of an already included sample^33–41^, 8 as they included mixed anxiety disorders containing patients with other than the defined DSM-5 anxiety diagnoses^42–49^, 2 articles because of younger aged samples^50,51^, 2 as they did not include a predefined outcome^52,53^, and 4 articles as they did not contain a predefined control group^54–57^. One additional study did not investigate a standardized ACT, MBCT, or MBSR program^58^. Thus, 23 RCTs published between 2007 and 2021 including 1815 patients were included in the meta-analysis^59–81^. Searching of trial registries revealed no additional unpublished or ongoing studies.

### Study characteristics

The characteristics of the included RCTs are presented in detail in Table 2. The RCTs were conducted in the US, Sweden, Canada, Germany, Norway, Brazil, Japan, Iran, Romania, and China and investigated patients diagnosed with Generalized Anxiety Disorder, Social Anxiety Disorder, and mixed anxiety diagnoses. Samples randomized ranged from 24 to 182 with a median N of 81 containing a median of 67.1% of women. The participants’ median age was 35.3 years.

**Table 2:** Characteristics of the included studies.

References	Origin	Sample	Mean age ± SD	Females	Sample rando-mized	Intervention			Concur-rent drug intake	Assessment	Outcomes included	Outcomes not included	Safety	Funding
						Treatment	Control							
Asmaee Majid 2012^59^	Iran	GAD	32.2 ± 2.2	0%	N = 33	8-week group MBSR + TAU	TAU		N.r	Post	Patient-rated anxiety (BAI) Depression (BDI-II)	Patient-rated anxiety (PSWQ)	N.r	No funding received
Boettcher 2014^60^	Sweden	PD, GAD, SAD, ADNOS	38.0 ± 10.3	71.4%	N = 91	8-week individual online MBSR + TAU	8-week individual online PE + TAU		26.3%	Post	Patient-rated anxiety (BAI)† Depression (BDI-II) Quality of life (QOLI)	None	N.r	University funding
Craske 2014^61^	United States	SAD	28.4 ± 6.8	46.0%	N = 100	12-week individual ACT + TAU	WL + TAU 12-week individual CBT + TAU		32.2%	Post 6mFU 12mFU	Clinician-rated anxiety (CSR)† Patient-rated social anxiety (composite score of LSAS-SR, SPS, SIAS)† Quality of life (QOLI)†	None	N.r	Governmental funding
Dahlin 2016^62^	Sweden	GAD	39.5 ± 10.7	83.5%	N = 103	9-week individual online ACT + TAU	WL + TAU		51.5%	Post	Patient-rated anxiety (BAI) Depression (MADRS) Quality of life (QOLI)	Patient-rated anxiety (PSWQ, GAD-7, GAD-Q-IV) Depression (PHQ9)	N.r	Governmental + university funding
de Almeida Sampaio 2020^63^	Brazil	GAD	36.5 ± 12.4	73.9%	N = 92	14-week group ABBT + TAU	14-week group PE + TAU		35.9%	Post 6mFU	Clinician-rated anxiety (HAM-A)† Patient-rated anxiety (DASS)† Depression (DASS) Quality of Life (WHOQOL)	Clinician-rated anxiety (CGI) Patient-rated anxiety (PSWQ)	N.r	N.r
Fathi 2017^64^	Iran	GAD	33.0 ± 4.7	100%	N = 40	12-week group ACT	WL		0%	Post 5mFU	Patient-rated anxiety (BAI)†	Patient-rated anxiety (MCQ-30)	N.r	No funding received
Gloster 2015^65^	Germany	PD, AP	36.9 ± 8.9	69.8%	N = 43	4-week individual ACT + TAU	WL + TAU		32.6%	Post	Clinician-rated anxiety (CGI)† Patient-rated anxiety (BAI)† Depression (BDI-II)‡	Patient-rated anxiety (PAS, MI, BSQ, ACQ, ASI, HAM-A, BAFT)	ACT: 0/33 AEs WL: 0/10 AEs	Governmental funding
Goldin 2016^66^	United States	SAD	32.7 ± 7.8	65.6%	N = 108	12-week group MBSR	WL 12-week group CBT		0%	Post 6mFU^§^ 12mFU^§^	Patient-rated anxiety (LSAS-SR)† Depression (RRS)	Patient-rated anxiety (SAFE)	N.r	Governmental funding
Hayes-Skelton 2013^67^	United States	GAD	32.9 ± 12.2	65.4%	N = 81	16-week individual ACT + TAU	16-week individual AR + TAU		18.6%	Post 10mFU	Clinician-rated anxiety (CSR)† Patient-rated anxiety (STAI-T)† Depression (BDI-II) Quality of life (QOLI)	Clinician-rated anxiety (HAM-A) Patient-rated anxiety (PSWQ, DASS)	ACT: 1/40 serious AE AR: 0/40 AEs	Governmental funding
Herbert 2018^68^	United States	SAD	30.0 ± 11.0	51.1%	N = 102	12-week individual ACT + TAU	12-week individual CBT + TAU		18%	Post	Clinician-rated anxiety (ADIS)† Patient-rated anxiety (LSAS-SR)† Depression (BDI) Quality of life (QOLI)	Clinician-rated anxiety (CGI) Patient-rated anxiety (SPAI, BAI) Quality of life (OQ-45)	N.r	No funding received
Hoge 2013^69^	United States	GAD	39.0 ± 13.0	51.0%	N = 93	8-week group MBSR + TAU	8-week group PE + TAU		15.1%	Post	Clinician-rated anxiety (HAM-A)† Patient-rated anxiety (BAI)	Clinician-rated anxiety (CGIS)	MBSR: 1/48 non-serious AE PE: 1/41 non-serious AE	Governmental + foundation funding
Ivanova 2016^70^	Sweden	SAD, PD	35.3 ± 11.0	64.5%	N = 152	10-week individual online ACT + TAU	WL + TAU		42.8%	Post	Patient-rated anxiety (LSAS-SR) Depression (PHQ-9) Quality of Life (QOLI)	Patient-rated anxiety (PDSS-SR; GAD-7†)	N.r	Governmental funding
Khoramnia 2020^71^	Iran	SAD	22.12 ± 1.08	70.8%	N = 24	12-week individual ACT	WL		0%	Post 5mFU	Patient-rated anxiety (SPIN)†	Patient-rated anxiety (AAQ-SA)	N.r	University funding
Kocovski 2013^72^	Canada	SAD	34.0 ± 11.1	55.5%	N = 137	12-week group ACT + TAU	WL + TAU 12-week group CBT + TAU		37.9%	Post 6mFU	Clinician-rated anxiety (LSAS-CA) Patient-rated anxiety (SPIN)† Depression (BDI-II)	Clinician-rated anxiety (CGI) Patient-rated anxiety (AAQ-SA) Depression (RRQ)	N.r	Governmental + foundation funding
Koszycki 2007^73^	Canada	SAD	38.2 ± 13.4	45.3%	N = 53	8-week group MBSR + TAU	12-week group CBT + TAU		28.3%	Post	Clinician-rated anxiety (LSAS-CA)† Patient-rated anxiety (SPS)† Depression (BDI-II) Quality of life (QOLI)	Clinician-rated anxiety (CGI) Patient-rated anxiety (SIAS, IPSM) Quality of life (LSRDS)	MBSR: 0/26 drop-out due to AE CBT: 2/27 drop-out due to AE	University funding
Koszycki 2016^75^	Canada	SAD	39.7 ± 15.5	79.0%	N = 39	12-week group MBSR + TAU	WL + TAU		23.1%	Post	Clinician-rated anxiety (LSAS-CA)† Patient-rated anxiety (SPIN)† Depression (BDI-II)	Clinician-rated anxiety (CGI) Patient-rated anxiety (SAS-SR)	MBSR: 1/21 unspecified AE WL: N.r	University funding
Koszycki 2021^74^	Canada	SAD	40.86 ± 13.74	62.9%	N = 97	12-week group MBSR + TAU	12-week group CBT + TAU		19.6%	Post 6mFU§	Clinician-rated anxiety (LSAS-CA)† Patient-rated anxiety (SPIN) Depression (BDI-II)	Patient-rated anxiety (SAS-SR)	MBSR: 2/52 drop-out due to AE CBT: 2/45 drop-out due to AE	Foundation funding
Ninomiya 2020^76^	Japan	PD, SAD	41.4 ± 10.0	37.5%	N = 40	8-week group MBCT + TAU	WL + TAU		95%	Post	Patient-rated anxiety (STAI-T)† Depression (CES-D) Quality of life (SF-12-PCS)	Patient-rated anxiety (LSAS, MIA, K6) Quality of life (EQ-5D)	MBSR: 0/20 serious AE WL: 0/20 serious AE	Govern-mental funding
Piet 2010^77^	Denmark	SAD	21.9 ± 2.7	68.5%	N = 26	8-week group MBCT + TAU	12-week group CBT + TAU		11.5%	Post	Clinician-rated anxiety (LSAS-CA)† Patient-rated anxiety (BAI) Depression (BDI-II) Quality of life (SDS)	Patient-rated anxiety (SPS, SIAS, FNE-BV)	MBCT: 1/8 non-serious AE CBT: 1/12 non-serious AE	N.r
Roemer 2008^78^	United States	GAD	33.59 ± 11.74	71.0%	N = 31	14-week individual ACT + TAU	WL + TAU		25.8%	Post	Clinician-rated anxiety (CSR)† Depression (BDI) Quality of life (QOLI)	Patient-rated anxiety (PSWQ, DASS, AAQ)	N.r	Governmental funding
Stefan 2019^79^	Romania	GAD	27.13 ± 7.5	84.5%	N = 75	16-week individual ABBT	16-week individual - CBT (BTP) - CBT (REBT)		0%	Post	Patient-rated anxiety (GAD-Q-IV)†	Patient-rated anxiety (PSWQ)†	N.r	Industrial funding
Vollestad 2011^80^	Norway	PD, SAD, GAD	42.5 ± 11.3	67.1%	N = 73	8-week group MBSR + TAU	WL + TAU		27.6%	Post	Patient-rated anxiety (BAI) Depression (BDI-II) Quality of life (SCL-90-R)	Clinician-rated anxiety (GSI) Patient-rated anxiety (STAI, PSWQ)	MBSR: 1/39 non-serious AE WL: 0/37 AE	N.r
Wong 2016^81^	China	GAD	50.0 ± 10.0	79.1%	N = 182	8-week group MBCT + TAU	WL + TAU 8-week group PE + TAU		33.5%	Post 5mFU 11mFU	Patient-rated anxiety (BAI)†‡ Depression (CES-D)‡ Quality of life (SF-12-PCS)‡	Patient-rated anxiety (PSWQ)	N.r	Governmental funding

AAQ-II = Acceptance and Action in Social Anxiety Questionnaire; ABBT = Acceptance-based Behavioral Therapy; ACQ = Agoraphobic Cognitions Questionnaire; ADIS: Anxiety Disorder interview Schedule; ADNOS = Anxiety Disorder Not Otherwise Specified; AP = Agoraphobia; AR = Applied Relaxation; ASI = Anxiety Sensitivity Index; BAI = Beck Anxiety Inventory; BAFT = Believability in Anxious Feelings and Thoughts Questionnaire; BDI-II = Beck Depression Inventory-II; BSQ = Bodily Sensations Questionnaire; CBT (BTP/REBT) = Cognitive Behavioral Therapy (Borkovec’s treatment package / Rational Emotive Behavior Therapy according to Ellis); CES-D = Centre for Epidemiological Studies-Depression Scale; CGI = Clinical Global Impression Severity Scale; CSR = Clinician Severity Ratings; DASS = Depression Anxiety and Stress Scale; EQ-5D = EuroQol 5-Dimension; FNE-BV = Fear of Negative Evaluation-Brief Version; GAD = Generalized Anxiety Disorder; GAD-Q-IV = Generalized Anxiety Disorder Questionnaire-IV; HAM-A = Hamilton Anxiety Rating Scale; IPSM = Interpersonal Sensitivity Measure; K6 = 6-item Psychological Distress Scale; LSAS-SR/CA = Liebowitz Social Anxiety Scale-Self Report/Clinician Administrated; LSRDS = Liebowitz Self-Rated Disability Scale; MADRS = Montgomery-Åsberg Depression Rating Scale; MBCT = Mindfulness Based Cognitive Therapy; MBSR = Mindfulness Based Stress Reduction; MCQ-30 = Metacognitions Questionnaire; MDD = Major Depressive Disorder; mFU = months after start of study; MIA = Mobility Inventory for Agoraphobia; N = sample size; N.r. = not reported; OQ-45 = 45-item Outcome Questionnaire; PAS = Panic Agoraphobia Scale; PD = Panic Disorder; PDSS-SR = Panic Disorder Severity Scale Self-Rated; PE: Psychoeducation; PHQ-9 = Patient Health Questionnaire; QOLI = Quality of Life Index; RRS = Ruminative Response Scale; RRQ = Rumination-Reflection Questionnaire; SAD = Social Anxiety Disorder; SAFE = Subtle Avoidance Frequency Examination; SAS-SR = Social Adjustment Scale Self-Report; SCL-90-R = Symptom Checklist-90-R; SDS = Shehan Disability Scale; SF-12-PCS = Short-Form Health Survey-Physical Component Score; SIAS = Social Interaction Anxiety Scale; SPIN = Social Phobia Inventory; SPS = Social Phobia Scale; STAI-T = State-Trait Anxiety Inventory-Trait Anxiety Subscale; TAU = treatment as usual; WHOQOL = World Health Organization Quality of Life Questionnaire; WL = wait-list; † = primary outcome(s) of the study; ‡ = data not published but provided by trial authors upon request; § = data not published & not provided by trial authors upon request.

Twelve RCTs investigated ACT interventions, 3 ones MBCT, and 8 ones MBSR. Individual- and group-based approaches varied as well as online and offline/in-person settings. Control interventions included TAU/wait-list, individualized or group-based CBT, psychoeducation, and relaxation. The median duration of the study treatments was 10 (4 to 16) weeks. Concurrent psychotropic drug use was reported by a median of 26.1% of the participants.

All studies provided data directly after the end of the intervention. Seven RCTs assessed a follow-up closest to 6 months after randomization, 4 RCTs additionally closest to 12 months after randomization. Outcomes included clinician-rated severity of anxiety, patient-rated severity of anxiety, patient-rated severity of depression, and patient-rated health-related quality of life.

Funding was reported by all but 3 RCTs and contained no specific funding (3 RCTs), university grants (4 RCTs), governmental grants (8 RCTs), university and governmental funding (1 RCT), private foundations (3 RCTs), and industrial funding (1 RCT).

### Risk of bias of individual studies

**Figure 2:**
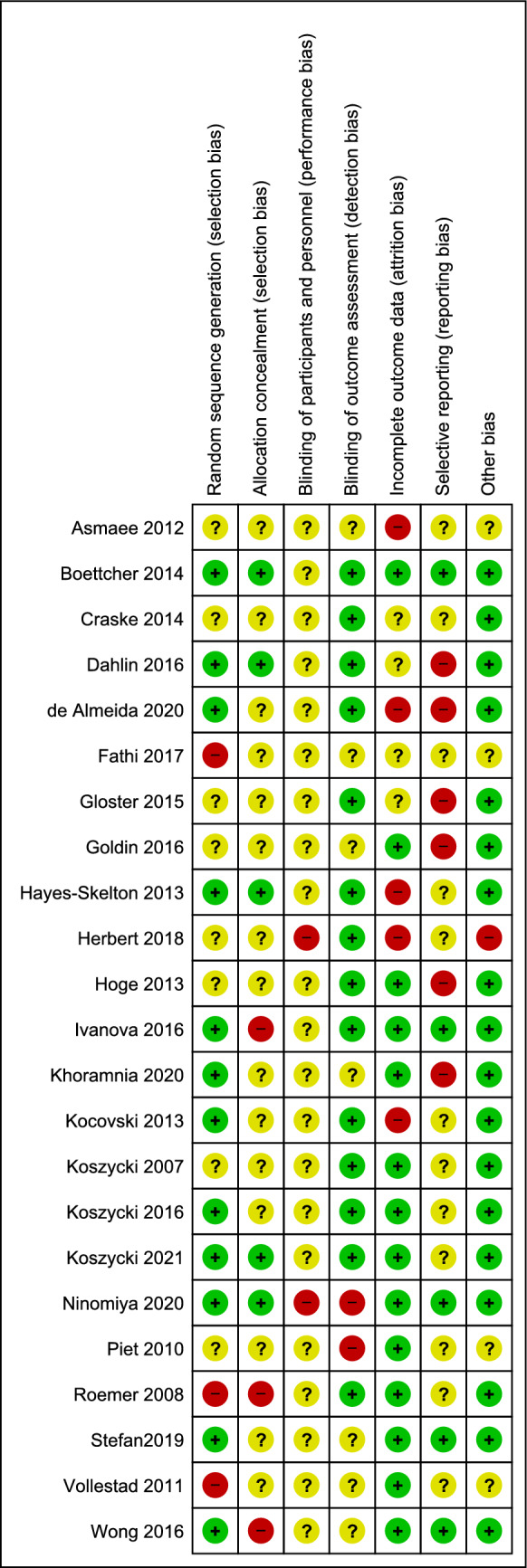
Risk of bias summary.

Risk of selection bias was assessed as low in 21.7% of the included studies. Additional 30.4% reported adequate random sequence generation but did not provide information or reported in-adequate information about allocation concealment. No study had low risk of performance bias. The risk of detection bias was low in 60.9% of the studies. Here, a low risk of bias judgement was also given in cases, when the outcome was clinician-reported and the clinician was blinded to group allocation but also when the staff who handed out the patient questionnaires was blinded, even if patients cannot be blinded. The same amount of the studies (60.9%) were assessed as low risk of attrition bias as they recorded less than 10% drop-outs per group or/and performed intention-to treat analysis. The risk of reporting bias and possible risks of other sources of bias were low in 21.7% and 78.3% of the studies, respectively (Fig. 2).

### Pooling of effects

#### Clinician-rated anxiety

**Figure 3:**
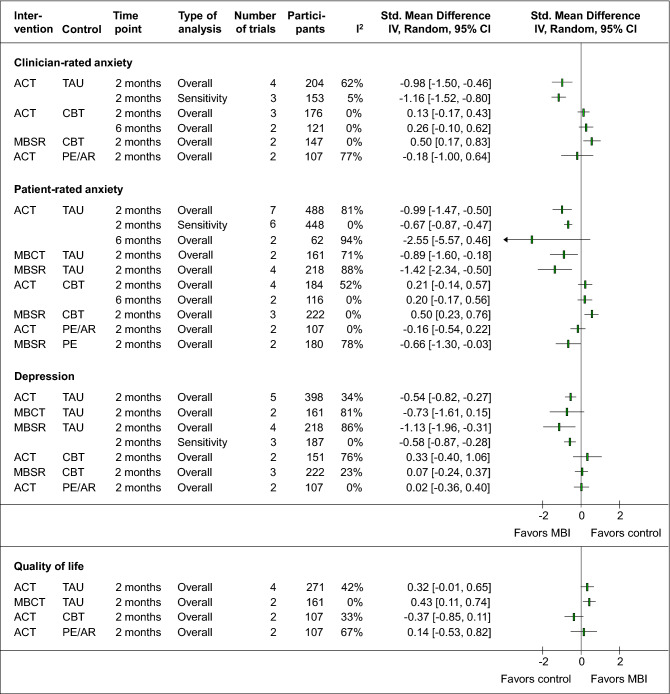
Forest plot summary of the effects on primary and secondary outcomes. Legend. ACT: Acceptance and Commitment Therapy; AR: Applied relaxation; CBT: Cognitive Behavioral Therapy; CI: Confidence interval; I^2^: Measure of statistical heterogeneity; IV: Inverse variance; MBCT: Mindfulness-based Cognitive Therapy; MBSR: Mindfulness-based stress reduction; PE: Psychoeducation; TAU: Treatment as usual.

ACT showed a significantly larger short-term effect than TAU on clinician-rated anxiety (4 RCTs, SMD = −0.98, 95%CI = [−1.50, −0.46], I^2^ = 62%, N = 204). The substantial heterogeneity could be reduced by excluding one RCT^65^ with mostly high/unclear risk of bias and the only effect which crossed zero (3 RCTs, SMD = −1.16, 95%CI = [−1.52, −0.80], I^2^ = 5%, N = 152) (Fig. 3). The analyses were robust against risk of detection bias, attrition bias, and other bias. In comparison to CBT, ACT homogeneously showed non-significant short-term (3 RCTs, SMD = 0.13, 95%CI = [−0.17, 0.43], I^2^ = 0%, N = 176) and medium-term effects up to 6 months (2 RCTs, SMD = 0.26, 95%CI = [−0.10, 0.62], I^2^ = 0%, N = 121) (Fig. 3). However, for both analyses, only the risk of detection bias and other bias was low. Up to 12 months, again no significant differences were found between ACT and CBT^61^. In comparison to less complex psychological interventions such as psychoeducation or relaxation, ACT did not show a significant larger effect (2 RCTs, SMD = −0.18, 95%CI = [−1.00, 0.64], I^2^ = 77%, N = 107) (Fig. 3). Because of the considerable heterogeneity, the pooled effect should be interpreted with restraint, also because one of the RCTs^63^ reported significant medium-term effects of ACT against psychoeducation. Detailed analyses can be found in the Supplementary Fig. 1.

One further RCT investigated MBSR against TAU and showed significantly higher improvements on clinician-rated anxiety up to 2 months post randomization^75^, while as compared to CBT, MBSR showed significantly lower short-term improvements (2 RCTs, SMD = 0.50, 95%CI = [0.17, 0.83], I^2^ = 0%, N = 147) (Fig. 1).

Further 2 RCTs of mostly unclear risk of bias, one on MBCT against CBT^77^ and another on MBSR against psychoeducation^69^ did not show any significant short-term superiority of the respective interventions.

#### Patient-rated anxiety

The meta-analyses of ACT in comparison to TAU revealed a significantly larger effect in favor of ACT (7 RCTs, SMD = −0.99, 95%CI = [−1.47, −0.50], I^2^ = 84%, N = 488) but revealed considerable heterogeneity, which could be decreased by excluding one RCT^64^ with the greatest effect in favor of ACT and the weakest study quality (6 RCTs, SMD = −0.67, 95%CI = [−0.87, −0.47], I^2^ = 0%, N = 448) (Fig. 3). The effect was robust against the risk of selection bias, detection bias, attrition bias, reporting bias, and other bias. No significant differences occurred for ACT versus TAU at 6 months (2 RCS, SMD = −2.55, 95%CI = [−5.57, 0.46], I^2^ = 94%, N = 62), while both individual trials^64,71^ reported significant differences. However, the two RCTs were both of unclear/high risk of bias and included very small samples, which may explain the considerable heterogeneity. The meta-analysis of ACT versus CBT contained RCTs with mostly unclear/high risk of bias and revealed neither significant short-term (4 RCTs, SMD = 0.21, 95%CI = [−0.14, 0.57], I^2^ = 52%, N = 284) nor medium-term (2 RCTs, SMD = 0.20, 95%CI = [−0.17, 0.56], I^2^ = 0%, N = 116) (Fig. 3) or longer-term differences^61^ between the two therapies. In comparison to less complex psychological interventions, ACT led to no significantly different short-term effect (2 RCTs, SMD = −0.16, 95%CI = [−0.54, 0.22], I^2^ = 0%, N = 107) (Fig. 3).

The meta-analyses of MBCT revealed a significant larger but heterogeneous short-term effect than TAU (2 RCTs, SMD = −0.89, 95%CI = [−160, −0.18], I^2^ = 71%, N = 161) (Fig. 3). At 6 months, a single RCT reported persisting longer-term effects as well^81^. In comparison to CBT, MBCT did not lead to significantly larger short-term effects^77^. MBCT and psychoeducation at 2, 6, and 12 months did not significantly differ from each other^81^.

The meta-analysis of MBSR versus TAU showed a significantly larger short-term effect in favor of MBSR (4 RCTs, SMD = −1.42, 95%CI = [−2.34, −0.50], I^2^ = 88%, N = 218) (Fig. 3). While the heterogeneity could not be reduced by excluding individual RCTs, the effect remained significant when excluding the RCTs with unclear/high risk of detection bias, attrition bias, and other bias. In comparison to CBT, the meta-analysis showed significantly lower improvements for MBSR (3 RCTs, SMD = 0.50, 95%CI = [0.23, 0.76], I^2^ = 0%, N = 222) (Fig. 1). In comparison to psychoeducation, MBSR showed a significantly larger short-term effect (2 RCTs, SMD = −0.66, 95%CI = [−1.30, −0.03], I^2^ = 78%, N = 180) (Fig. 3). The meta-analysis contained considerable heterogeneity but could be considered as robust against all risk of bias domains except the risk of performance bias. Detailed analyses on patient-rated anxiety is provided in Supplementary Fig. 2.

#### Depressive symptoms

For depressive symptoms, the meta-analysis revealed significantly higher short-term effects of ACT than of TAU (5 RCTs, SMD = −0.54, 95%CI = [−0.82, −0.27], I^2^ = 34%, N = 398), which was found to be robust against risk of selection bias, detection bias, and other bias. In comparison to CBT (2 RCTs, SMD = 0.33, 95%CI = [−0.40, 1.06], I^2^ = 76%, N = 151) and less complex psychological interventions (2 RCTs, SMD = 0.02, 95%CI = [−0.36, 0.40], I^2^ = 0%, N = 107), ACT did not reveal larger short-term effects (Fig. 3). Longer-term effects at 12 months did also not significantly differ from psychoeducation^67^.

For MBCT, the meta-analysis did not show significantly higher effects on depressive symptoms against TAU, neither at short-term (2 RCTs, SMD = −0.73, 95%CI = [−1.61, 0.15], I^2^ = 81%, N = 161) nor at 6 months^81^. Short- and longer-term effects did not differ between MBCT and CBT^77^ or psychoeducation^81^.

For MBSR, the meta-analysis revealed significantly larger short-term anti-depressive effects against TAU (4 RCTs, SMD = −1.13, 95%CI = [−1.96, −0.31], I^2^ = 86%, N = 218). The considerable heterogeneity could be reduced by excluding one low-quality RCT with the largest effect that compared MBSR to TAU without using a waiting list^59^. This resulted in still significantly higher short-term effects of MBSR compared to TAU (3 RCTs, SMD = −0.58, 95%CI = [−0.87, −0.28], I^2^ = 0%, N = 187) (Fig. 3). The effect was robust against risk of detection bias, attrition bias, and other bias. In comparison to CBT, no superiority of MBSR on depressive symptoms could be detected (3 RCTs, SMD = 0.07, 95%CI = [−0.24, 0.37], I^2^ = 23%, N = 222) (Fig. 3). One additional RCT showed that MBSR was superior to psychoeducation in reducing short-term depressive symptoms^60^. Detailed analyses on depression can be found in the Supplementary Fig. 3.

#### Quality of life

ACT showed no significantly different short-term effect on quality of life compared to TAU (4 RCTs, SMD = 0.32, 95%CI = [−0.01, 0.65], I^2^ = 42%, N = 271), CBT (2 RCTs, SMD = −0.37, 95%CI = [−0.85, 0.11], I^2^ = 33%, N = 107) or less complex psychotherapeutic interventions (2 RCTs, SMD = 0.14, 95%CI = [−0.53, 0.82], I^2^ = 67%, N = 107) (Fig. 3). Longer-term effects of ACT did not significantly differ from those of CBT at 6 and 12 months^61^, were reported as significantly higher as those of psychoeducation at 6 months^63^, but not significantly different from those of relaxation at 12 months^67^.

MBCT was found to be superior to TAU in the short-term (2 RCTs, SMD = 0.43, 95%CI = [0.11, 0.74], I^2^ = 0%, N = 161) (Fig. 3), but not to CBT^77^ or to psychoeducation^81^. More detailed analyses can be found in the Supplementary Fig. 4.

Further RCTs reported significantly higher short-term effects on quality of life in favor of MBSR compared to TAU^80^ but not compared to CBT^73^ or psychoeducation^60^.

### Safety

Safety data were reported insufficiently (Tab. 2). Fourteen RCTs did not report any information on AEs or reasons for study withdrawal. Serious AEs were reported by one RCT in the ACT group (bypass surgery), which was highly likely not related to the study intervention^67^. Minor AEs were equally distributed between experimental and control groups^65,69,73–77,80^.

## Discussion

### Summary of evidence

The literature search revealed 23 RCTs investigating the effectiveness of ACT, MBCT, and MBSR in patients with DSM-5 anxiety disorders such as Generalized Anxiety Disorder, Social Anxiety Disorder, and mixed samples including different anxiety diagnoses. The meta-analyses revealed at least short-term effects on clinician- and patient-rated anxiety for ACT, MBCT and MBSR in addition to TAU versus TAU alone. In comparison to CBT, ACT and MBCT showed comparable effects on both anxiety outcomes, while MBSR showed significantly lower effects. Pooled effects up to 6 months post randomization can only be calculated for ACT but did not show any significant differences compared to TAU or CBT. Short-term effects of ACT, MBCT and MBSR on secondary outcomes were superior against TAU but not against CBT or less complex psychotherapeutic interventions such as psychoeducation or applied relaxation. Most effects were robust against most risk of bias domains. However, the risk of selection bias and performance bias remains unclear or high for almost all meta-analyses. Statistical heterogeneity could be reduced in several meta-analyses by excluding low-quality studies with extreme values. A correlation of those extreme values with funding concerns from industrial companies or private foundations could not be determined. Moreover, comparisons to CBT should be interpreted with restraint as the number of included trials was very low and none of the studies tested non-inferiority. Adverse events were reported insufficiently. If safety issues were reported, ACT, MBCT, and MBSR did not lead to more adverse events than usual care and comparable adverse events to CBT.

In contrast to prior systematic reviews, which found promising effects of mindfulness on anxiety symptoms in the general population^13,14,23^ and somatically ill samples^15,17^, the evidence for patients with manifest DSM-5 anxiety disorders should be interpreted with restraint. Reasons include the low number of studies investigating longer-term effects against eligible control conditions such as CBT, the overall unclear study quality, and the often missed systematic assessment and reporting of adverse events.

### Limitations

This meta-analysis has further limitations. We often were not able to pool data of RCTs because of non-comparable controls, or could perform sensitivity analyses of comparisons including only 2 RCTs. In addition, we were not able to calculate subgroup analyses of different anxiety disorders. Thus, the present meta-analysis does not allow to draw conclusions for a specific diagnosis. Since risk of publication bias could not be assessed, the effects might also be overestimated due to unpublished studies, even if the search of trial registries did not result in registered but unpublished studies^82^.

### Implications for further research

Further higher-quality trials on mindfulness-based interventions are needed to verify the effects in patients with manifest anxiety disorders. Authors should ensure rigorous methodology and reporting according to CONSORT^83^ and choose adequate control conditions. Recent meta-analyses on pharmacological and psychological intervention for anxiety disorders conclude that wait-list control groups may produce nocebo effects in trials of psychotherapy^84^. TAU, in addition, was also found to be a very heterogeneous control condition and anything but usual or standard care^85,86^. Psychological placebo effects were estimated on average 0.83 in patients with anxiety disorders^87^. Thus, adequate control groups for trials on manifest disorders rather than subclinical symptoms should be designed considering nocebo as well as placebo effects. Non-inferiority trials against standard psychotherapies such as CBT are missing completely. To enhance study quality and reduce the risk of performance bias, even if patients and therapists cannot be blinded, controlling for patients’ treatment expectations and their perceptions of quality of the alliance towards the treating therapist would be feasible. Blinding of outcome assessors, especially for clinician-rated outcomes as well as more adequate statistics (including intention-to-treat analyses) should be standard. Future studies are requested to strictly report AEs and reasons of drop-out.

## Conclusions

The evidence suggests clinically relevant short-term anxiolytic effects of acceptance-based and with less extent of mindfulness-based interventions when added to usual care that, however, might be a result of nocebo- and/or placebo effects. The relevance of longer-term effects as well as the comparability to standard CBT remain preliminary until further high-quality studies will be published.

## Supplementary Information

Supplementary Information.

## Data Availability

The data and material analyzed during the current study are available from the corresponding author on request.

## Code availability

The RevMan code used for the current study is available from the corresponding author on request.
