# Supplementary material for: A systematic review and meta-analysis of acceptance- and mindfulness-based interventions for DSM-5 anxiety disorders
Source: Sci Rep. 2021 Oct 14;11:20385. doi: 10.1038/s41598-021-99882-w (PMC8516851; doi:10.1038/s41598-021-99882-w)
Supplement: Supplementary file 1 — Supplementary Information. [file 41598_2021_99882_MOESM1_ESM.pdf]

Acceptance- and mindfulness-based interventions for DSM-5 anxiety disorders: a systematic review and meta-analysis

Heidemarie Haller, Pascal Breilmann, Marleen Schröter, Gustav Dobos, Holger Cramer
Department of Internal and Integrative Medicine, Evang. Kliniken Essen-Mitte, Faculty of Medicine, University of Duisburg-Essen, Germany
E-mail: h.haller@kem-med.com

	MBI			Control				Std. Mean Difference
**Outcome/Study**	**Mean**	**SD**	**Total**	**Mean**	**SD**	**Total**	**Weight**	**IV, Random, 95% CI**
**1. Clinician-rated anxiety**								
**1.1. ACT versus TAU**								
**1.1.1. Two months**								
Craske 2014	3.6	1.315	29	5.3	1.4536	25	26.9%	-1.21 [-1.80, -0.63]
Gloster 2015	14	10.8	41	16.3	10.5	10	23.6%	-0.21 [-0.90, 0.48]
Kocovski 2013	49.64	19.87	37	68.53	20.04	31	29.5%	-0.94 [-1.44, -0.43]
Roemer 2008	3.18	1.162	15	5.32	1.36	16	19.9%	-1.64 [-2.47, -0.81]
**Overall analysis (95% CI)**			**122**			**82**	**100.0%**	**-0.98 [-1.50, -0.46]**
Heterogeneity: Tau² = 0.17; Chi² = 7.83, df = 3 (P = 0.05); I² = 62%								
Test for overall effect: Z = 3.72 (P < 0.001)								
**Sensitivity analysis (95% CI) without Gloster 2015**			**81**			**72**	**76.5%**	**-1.16 [-1.52, -0.80]**
Heterogeneity: Tau² = 0.00; Chi² = 2.09, df = 2 (P = 0.35); I² = 5%								
Test for overall effect: Z = 6.38 (P < 0.001)								
**1.2. ACT versus CBT**								
**1.2.1. Two months**								
Craske 2014	3.6	1.32	29	3.2	1.41	33	35.5%	0.29 [-0.21, 0.79]
Herbert 2018	30.7	9.72	28	29.44	13.47	17	24.6%	0.11 [-0.49, 0.71]
Kocovski 2013	49.64	19.87	37	49.48	21.95	32	39.9%	0.01 [-0.47, 0.48]
**Overall analysis (95% CI)**			**94**			**82**	**100.0%**	**0.13 [-0.17, 0.43]**
Heterogeneity: Tau² = 0.00; Chi² = 0.65, df = 2 (P = 0.72); I² = 0%								
Test for overall effect: Z = 0.87 (P = 0.38)								
**1.2.2. Six months**								
Craske 2014	3.5	1.32	29	3.2	1.41	33	51.5%	0.22 [-0.28, 0.72]
Kocovski 2013	49.28	18.96	32	43.52	18.91	27	48.5%	0.30 [-0.22, 0.82]
**Overall analysis (95% CI)**			**61**			**60**	**100.0%**	**0.26 [-0.10, 0.62]**
Heterogeneity: Tau² = 0.00; Chi² = 0.05, df = 1 (P = 0.82); I² = 0%								
Test for overall effect: Z = 1.40 (P = 0.16)								
**1.3. ACT versus PE / AR**								
**1.3.1. Two months**								
de Almeida 2020	11.81	6.54	21	16.17	7.33	23	47.7%	-0.61 [-1.22, -0.01]
Hayes-Skelton 2013	3.03	1.38	30	2.7	1.57	33	52.3%	0.22 [-0.28, 0.72]
**Overall analysis (95% CI)**			**51**			**56**	**100.0%**	**-0.18 [-1.00, 0.64]**
Heterogeneity: Tau² = 0.27; Chi² = 4.36, df = 1 (P = 0.04); I² = 77%								
Test for overall effect: Z = 0.43 (P = 0.67)								
**1.4. MBSR versus CBT**								
**1.4.1. Two months**								
Koszycki 2007	28.90	8.90	26	23.0	9.50	27	35.6%	0.63 [0.08, 1.18]
Koszycki 2021	59.5	22.44	52	50.1	21.1	42	64.4%	0.43 [0.02, 0.84]
**Overall analysis (95% CI)**			**78**			**69**	**100.0%**	**0.50 [0.17, 0.83]**
Heterogeneity: Tau² = 0.00; Chi² = 0.34, df = 1 (P = 0.56); I² = 0%								
Test for overall effect: Z = 2.97 (P = 0.003)								

Std. Mean Difference IV, Random, 95% CI

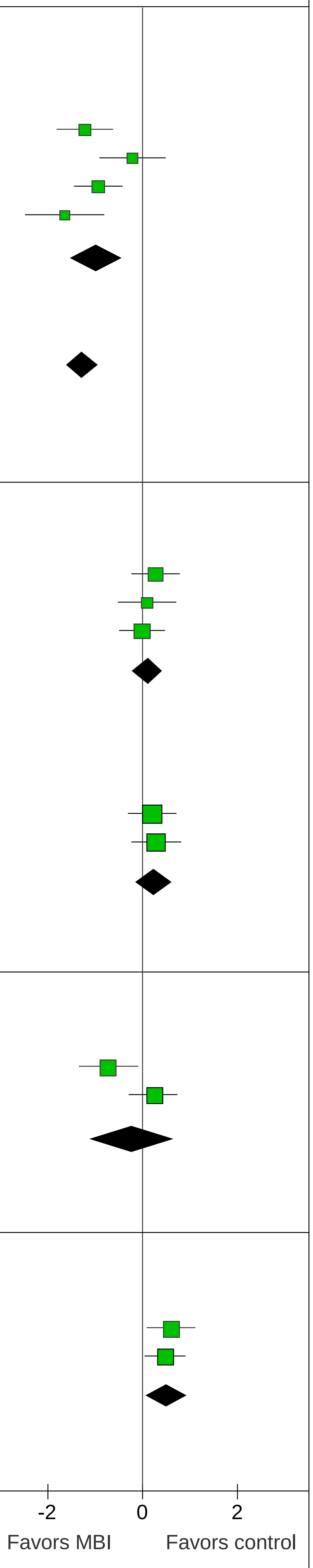

Supplementary Fig. 1 Forest plot of the effects on clinician-rated anxiety. Legend. ACT: Acceptance and Commitment Therapy; AR: Applied relaxation; CBT: Cognitive Behavioral Therapy; CI: Confidence interval; I²: Measure of statistical heterogeneity; IV: Inverse variance; PE: Psychoeducation; TAU: Treatment as usual

Outcome/Study	MBI Mean	MBI SD	MBI Total	Control Mean	Control SD	Control Total	Weight	Std. Mean Difference IV, Random, 95% CI
**2. Patient-rated anxiety**								
**2.1. ACT versus TAU**								
**2.1.1. Two months**								
Craske 2014	-1	1.052	29	-0.3	0.969	25	15.2%	-0.68 [-1.23, -0.13]
Dahlin 2016	12.67	8.24	42	17.09	7.78	43	16.4%	-0.55 [-0.98, -0.11]
Fathi 2017	12.73	1.87	20	32.69	7.18	20	9.9%	-3.73 [-4.79, -2.67]
Gloster 2015	3.5	1	41	4.1	0.7	10	13.5%	-0.62 [-1.32, 0.08]
Ivanova 2016	52.974	22.849	101	68.98	22.73	51	17.2%	-0.70 [-1.04, -0.35]
Khoramnia 2020	46.6	19.45	11	61.45	10.89	11	11.6%	-0.91 [-1.79, -0.02]
Kocovski 2013	33.91	14.79	53	43.28	9.9	31	16.2%	-0.70 [-1.16, -0.25]
**Overall analysis (95% CI)**			**297**			**191**	**100.0%**	**-0.99 [-1.47, -0.50]**
Heterogeneity: Tau² = 0.32; Chi² = 31.54, df = 6 (P < 0.001); I² = 81%								
Test for overall effect: Z = 3.99 (P < 0.0001)								
**Sensitivity analysis (95% CI) without Fathi 2017**			**277**			**171**	**90.1%**	**-0.67 [-0.87, -0.47]**
Heterogeneity: Tau² = 0.00; Chi² = 0.65, df = 5 (P = 0.99); I² = 0%								
Test for overall effect: Z = 6.56 (P < 0.0001)								
**2.1.2. Six months**								
Fathi 2017	12.2	1.93	20	32.77	6.66	20	49.4%	-4.11 [-5.25, -2.98]
Khoramnia 2020	49.77	13.47	11	62.27	9.44	11	50.6%	-1.03 [-1.94, -0.13]
**Overall analysis (95% CI)**			**31**			**31**	**100.0%**	**-2.55 [-5.57, 0.46]**
Heterogeneity: Tau² = 4.46; Chi² = 17.36, df = 1 (P < 0.0001); I² = 94%								
Test for overall effect: Z = 1.66 (P = 0.10)								
**2.2. MBCT versus TAU**								
**2.2.1. Two months**								
Ninomiya 2020	42.5	11	20	57.6	11.5	20	41.7%	-1.32 [-2.01, -0.63]
Wong 2016	25.98	10.93	61	32.98	12.97	60	58.3%	-0.58 [-0.94, -0.22]
**Overall analysis (95% CI)**			**81**			**80**	**100.0%**	**-0.89 [-1.60, -0.18]**
Heterogeneity: Tau² = 0.19; Chi² = 3.41, df = 1 (P = 0.06); I² = 71%								
Test for overall effect: Z = 2.45 (P = 0.01)								
**2.3. MBSR versus TAU**								
**2.3.1. Two months**								
Asmaee 2012	12.93	3.71	16	28.31	7.15	15	21.7%	-2.66 [-3.65, -1.66]
Goldin 2016	54.8	18.6	36	76.2	22.8	36	26.9%	-1.02 [-1.51, -0.52]
Koszycki 2016	23.22	7.8	21	41.0	9.6	18	24.1%	-2.01 [-2.79, -1.22]
Vollestad 2011	12.2	10.5	39	15.5	10.1	37	27.3%	-0.32 [-0.77, 0.14]
**Overall analysis (95% CI)**			**112**			**106**	**100.0%**	**-1.42 [-2.34, -0.50]**
Heterogeneity: Tau² = 0.76; Chi² = 25.64, df = 3 (P < 0.001); I² = 88%								
Test for overall effect: Z = 3.03 (P = 0.002)								
**2.4. ACT versus CBT**								
**2.4.1. Two months**								
Craske 2014	-1	1.05	29	-1.1	0.85	33	24.7%	0.10 [-0.40, 0.60]
Herbert 2018	54.14	19.17	28	36	20.07	17	19.0%	0.91 [0.28, 1.55]
Kocovski 2013	33.91	14.79	53	33.72	14.07	53	31.1%	0.01 [-0.37, 0.39]
Stefan 2019	6.29	3.18	24	6.145	3.805	47	25.1%	0.04 [-0.45, 0.53]
**Overall analysis (95% CI)**			**134**			**150**	**100.0%**	**0.21 [-0.14, 0.57]**
Heterogeneity: Tau² = 0.07; Chi² = 6.27, df = 3 (P = 0.10); I² = 52%								
Test for overall effect: Z = 1.18 (P = 0.24)								
**2.4.2. Six months**								
Craske 2014	-1.1	0.79	29	-1.2	1.13	33	53.9%	0.10 [-0.40, 0.60]
Kocovski 2013	29.4	13.72	30	25.33	12.11	24	46.1%	0.31 [-0.23, 0.85]
**Overall analysis (95% CI)**			**59**			**57**	**100.0%**	**0.20 [-0.17, 0.56]**
Heterogeneity: Tau² = 0.00; Chi² = 0.31, df = 1 (P = 0.58); I² = 0%								
Test for overall effect: Z = 1.05 (P = 0.30)								
**2.5. MBSR versus CBT**								
**2.5.1. Two months**								
Goldin 2016	54.8	18.6	36	49.0	16.3	36	33.2%	0.33 [-0.14, 0.79]
Koszycki 2007	24.60	16.40	26	15.4	8.0	27	23.2%	0.71 [0.15, 1.26]
Koszycki 2021	31.14	11.93	52	25.35	10.37	45	43.6%	0.51 [0.11, 0.92]
**Overall analysis (95% CI)**			**114**			**108**	**100.0%**	**0.50 [0.23, 0.76]**
Heterogeneity: Tau² = 0.00; Chi² = 1.06, df = 2 (P = 0.59); I² = 0%								
Test for overall effect: Z = 3.63 (P = 0.0003)								
**2.6. ACT versus PE / AR**								
**2.6.1. Two months**								
de Almeida 2020	6.48	6.84	21	9.39	7.64	23	40.6%	-0.39 [-0.99, 0.20]
Hayes-Skelton 2013	43.46	10.39	30	43.48	12.07	33	59.4%	-0.00 [-0.50, 0.49]
**Overall analysis (95% CI)**			**51**			**56**	**100.0%**	**-0.16 [-0.54, 0.22]**
Heterogeneity: Tau² = 0.00; Chi² = 0.98, df = 1 (P = 0.32); I² = 0%								
Test for overall effect: Z = 0.83 (P = 0.41)								
**2.7. MBSR versus PE**								
**2.7.1. Two months**								
Boettcher 2014	11.8	7.8	45	20.8	10	46	49.6%	-0.99 [-1.43, -0.56]
Hoge 2013	9.1	7.11	48	11.33	5.65	41	50.4%	-0.34 [-0.76, 0.08]
**Overall analysis (95% CI)**			**93**			**87**	**100.0%**	**-0.66 [-1.30, -0.03]**
Heterogeneity: Tau² = 0.17; Chi² = 4.46, df = 1 (P = 0.03); I² = 78%								
Test for overall effect: Z = 2.04 (P = 0.04)								

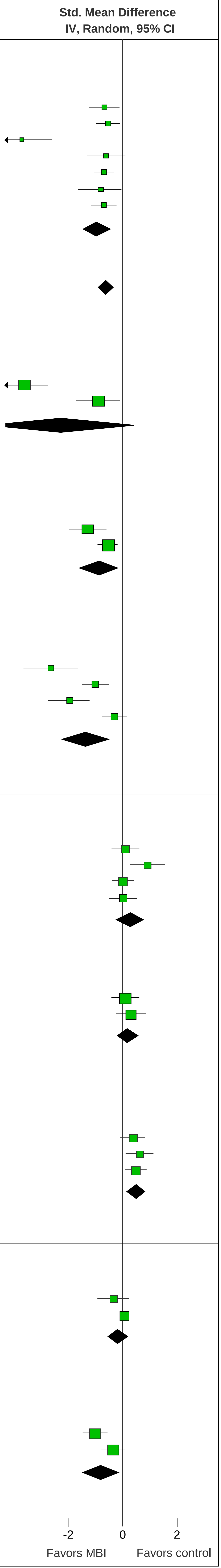

Supplementary Fig. 2 Forest plot of the effects on patient-rated anxiety. Legend. ACT: Acceptance and Commitment Therapy; AR: Applied relaxation; CBT: Cognitive Behavioral Therapy; CI: Confidence interval; I²: Measure of statistical heterogeneity; IV: Inverse variance; MBCT: Mindfulness-based Cognitive Therapy; MBSR: Mindfulness-based stress reduction; PE: Psychoeducation; TAU: Treatment as usual

Outcome/Study	MBI Mean	MBI SD	MBI Total	Control Mean	Control SD	Control Total	Weight	Std. Mean Difference IV, Random, 95% CI
**3. Depression**								
**3.1. ACT versus TAU**								
**3.1.1. Two months**								
Dahlin 2016	12.17	6.89	42	15.79	5.97	43	23.9%	-0.56 [-0.99, -0.12]
Gloster 2015	8.06	10	36	14.8	12.3	10	11.8%	-0.63 [-1.34, 0.08]
Ivanova 2016	5.797	4.906	101	7.4	5	51	31.3%	-0.32 [-0.66, 0.02]
Kocovski 2013	12.41	8.74	53	16.32	9.89	31	23.0%	-0.42 [-0.87, 0.03]
Roemer 2008	5.77	5.1511	15	16.52	9.32	16	9.9%	-1.38 [-2.17, -0.58]
**Overall analysis (95% CI)**			**247**			**151**	**100.0%**	**-0.54 [-0.82, -0.27]**
Heterogeneity: Tau² = 0.03; Chi² = 6.05, df = 4 (P = 0.20); I² = 34%								
Test for overall effect: Z = 3.89 (P = 0.0001)								
**3.2. MBCT versus TAU**								
**3.2.1. Two months**								
Ninomiya 2020	12.6	7.6	20	23.6	9.8	20	44.6%	-1.23 [-1.91, -0.55]
Wong 2016	44.4	10.76	61	47.94	10.67	60	55.4%	-0.33 [-0.69, 0.03]
**Overall analysis (95% CI)**			**81**			**80**	**100.0%**	**-0.73 [-1.61, 0.15]**
Heterogeneity: Tau² = 0.33; Chi² = 5.26, df = 1 (P = 0.02); I² = 81%								
Test for overall effect: Z = 1.63 (P = 0.10)								
**3.3. MBSR versus TAU**								
**3.3.1. Two months**								
Asmaee 2012	14.13	3.64	16	30.5	5.77	15	21.3%	-3.33 [-4.46, -2.20]
Goldin 2016	11.9	4.2	36	13.7	3.3	36	27.0%	-0.47 [-0.94, -0.00]
Koszycki 2016	4.83	5.7	21	9.67	6.2	18	24.2%	-0.80 [-1.46, -0.14]
Vollestad 2011	10.8	9.9	39	16.2	8.9	37	27.4%	-0.57 [-1.03, -0.11]
**Overall analysis (95% CI)**			**112**			**106**	**100.0%**	**-1.13 [-1.96, -0.31]**
Heterogeneity: Tau² = 0.58; Chi² = 21.91, df = 3 (P < 0.0001); I² = 86%								
Test for overall effect: Z = 2.69 (P = 0.007)								
**Sensitivity analysis (95% CI) without Asmaee 2012**			**96**			**91**	**78.7%**	**-0.58 [-0.87, -0.28]**
Heterogeneity: Tau² = 0.00; Chi² = 0.63, df = 2 (P = 0.73); I² = 0%								
Test for overall effect: Z = 3.85 (P < 0.001)								
**3.4. ACT versus CBT**								
**3.4.1 Two months**								
Herbert 2018	11.41	9.15	28	4.94	7.21	17	44.4%	0.75 [0.13, 1.37]
Kocovski 2013	12.41	8.74	53	12.45	9.64	53	55.6%	-0.00 [-0.39, 0.38]
**Overall analysis (95% CI)**			**81**			**70**	**100.0%**	**0.33 [-0.40, 1.06]**
Heterogeneity: Tau² = 0.21; Chi² = 4.09, df = 1 (P = 0.04); I² = 76%								
Test for overall effect: Z = 0.88 (P = 0.38)								
**3.5. MBSR versus CBT**								
**3.5.1. Two months**								
Goldin 2016	11.9	4.2	36	10.4	3.9	36	32.8%	0.37 [-0.10, 0.83]
Koszycki 2007	9.1	7.5	26	9.0	9.8	27	26.0%	0.01 [-0.53, 0.55]
Koszycki 2021	7.45	8.7	52	8.65	9.0	45	41.2%	-0.13 [-0.53, 0.26]
**Overall analysis (95% CI)**			**114**			**108**	**100.0%**	**0.07 [-0.24, 0.37]**
Heterogeneity: Tau² = 0.02; Chi² = 2.60, df = 2 (P = 0.27); I² = 23%								
Test for overall effect: Z = 0.43 (P = 0.66)								
**3.6. ACT versus PE / AR**								
**3.6.1 Two months**								
de Almeida 2020	9.52	11.12	21	11.57	8.82	23	41.1%	-0.20 [-0.79, 0.39]
Hayes-Skelton 2013	9.54	10.76	30	7.85	8.51	33	58.9%	0.17 [-0.32, 0.67]
**Overall analysis (95% CI)**			**51**			**56**	**100.0%**	**0.02 [-0.36, 0.40]**
Heterogeneity: Tau² = 0.00; Chi² = 0.90, df = 1 (P = 0.34); I² = 0%								
Test for overall effect: Z = 0.10 (P = 0.92)								

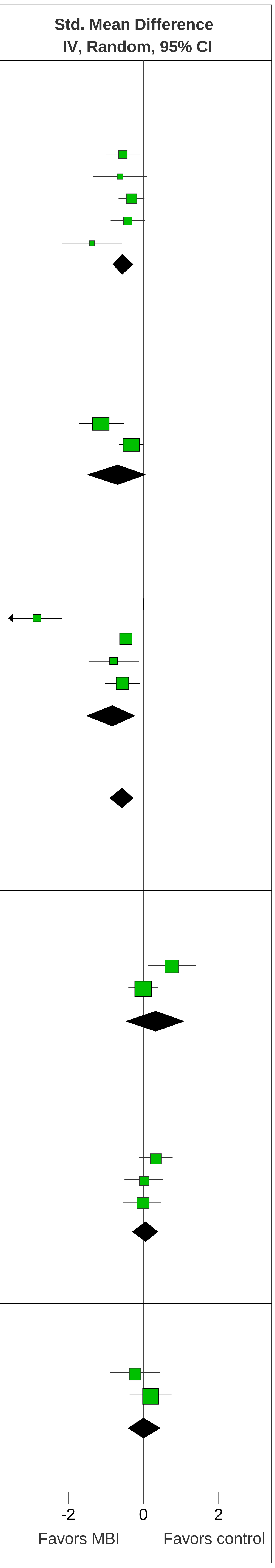

Supplementary Fig. 3 Forest plot of the effects on depression. Legend. ACT: Acceptance and Commitment Therapy; AR: Applied relaxation; CBT: Cognitive Behavioral Therapy; CI: Confidence interval; I^2: Measure of statistical heterogeneity; IV: Inverse variance; MBCT: Mindfulness-based Cognitive Therapy; MBSR: Mindfulness-based stress reduction; PE: Psychoeducation; TAU: Treatment as usual

Outcome/Study	MBI Mean	MBI SD	MBI Total	Control Mean	Control SD	Control Total	Weight	Std. Mean Difference IV, Random, 95% CI
**4. Quality of life**								
**4.1. ACT versus TAU**								
**4.1.1. Two months**								
Craske 2014	0.5	1.84	29	0.1	1.696	25	23.2%	0.22 [-0.31, 0.76]
Dahlin 2016	1.68	1.33	42	1.51	1.4	43	30.0%	0.12 [-0.30, 0.55]
Ivanova 2016	1.21	1.7	50	0.84	1.57	51	32.6%	0.22 [-0.17, 0.62]
Roemer 2008	2	1.743	15	0.16	1.48	16	14.2%	1.11 [0.35, 1.88]
**Overall analysis (95% CI)**			**136**			**135**	**100.0%**	**0.32 [-0.01, 0.65]**
Heterogeneity: Tau² = 0.05; Chi² = 5.19, df = 3 (P = 0.16); I² = 42%								
Test for overall effect: Z = 1.90 (P = 0.06)								
**4.2. MBCT versus TAU**								
**4.2.1. Two months**								
Ninomiya 2020	55.3	8.1	20	48.9	13.8	20	24.4%	0.55 [-0.08, 1.19]
Wong 2016	40.29	7.36	61	37.47	7.22	60	75.6%	0.38 [0.02, 0.74]
**Subtotal (95% CI)**			**81**			**80**	**100.0%**	**0.43 [0.11, 0.74]**
Heterogeneity: Tau² = 0.00; Chi² = 0.21, df = 1 (P = 0.65); I² = 0%								
Test for overall effect: Z = 2.67 (P = 0.008)								
**4.3. ACT versus CBT**								
**4.3.1. Two months**								
Craske 2014	0.5	1.84	29	0.8	1.97	33	57.1%	-0.16 [-0.65, 0.34]
Herbert 2018	0.19	0.143	28	0.92	1.8	17	42.9%	-0.65 [-1.27, -0.03]
**Overall analysis (95% CI)**			**57**			**50**	**100.0%**	**-0.37 [-0.85, 0.11]**
Heterogeneity: Tau² = 0.04; Chi² = 1.49, df = 1 (P = 0.22); I² = 33%								
Test for overall effect: Z = 1.50 (P = 0.13)								
**4.4. ACT versus PE / AR**								
**4.4.1. Two months**								
de Almeida 2020	64.63	14.86	21	56.5	16.24	23	46.8%	0.51 [-0.09, 1.11]
Hayes-Skelton 2013	1.56	1.78	30	1.87	1.6	33	53.2%	-0.18 [-0.68, 0.31]
**Subtotal (95% CI)**			**51**			**56**	**100.0%**	**0.14 [-0.53, 0.82]**
Heterogeneity: Tau² = 0.16; Chi² = 3.04, df = 1 (P = 0.08); I² = 67%								
Test for overall effect: Z = 0.41 (P = 0.68)								

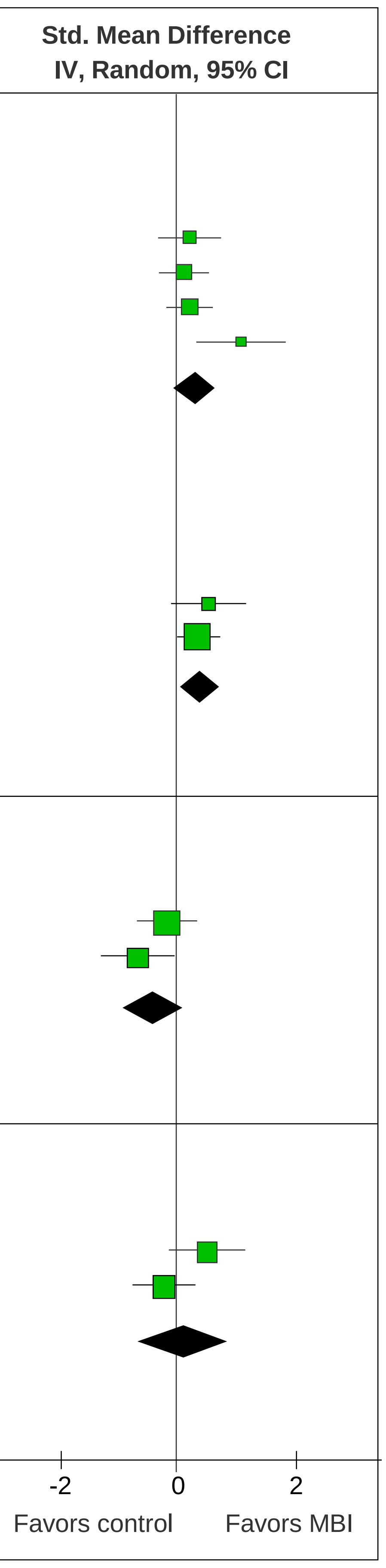

Supplementary Fig. 4 Forest plot of the effects on quality of life. Legend. ACT: Acceptance and Commitment Therapy; AR: Applied relaxation; CBT: Cognitive Behavioral Therapy; CI: Confidence interval; I^2: Measure of statistical heterogeneity; IV: Inverse variance; MBCT: Mindfulness-based Cognitive Therapy; PE: Psychoeducation; TAU: Treatment as usual
